# Effect of Epstein–Barr Virus Infection on Selected Immunological Parameters in Children with Type 1 Diabetes

**DOI:** 10.3390/ijms24032392

**Published:** 2023-01-25

**Authors:** Maria Klatka, Izabela Rysz, Anna Hymos, Agnieszka Polak, Paulina Mertowska, Sebastian Mertowski, Konrad Smolak, Ewelina Grywalska

**Affiliations:** 1 Department of Pediatric Endocrinology and Diabetology, Medical University of Lublin, 20-093 Lublin, Poland; 2 Department of Experimental Immunology, Medical University of Lublin, 20-093 Lublin, Poland; 3 Department of Endocrinology, Medical University of Lublin, 20-093 Lublin, Poland

**Keywords:** Epstein–Barr virus, type 1 diabetes, children, immune system

## Abstract

Diabetes mellitus is a group of metabolic disorders with different etiologies, pathogeneses and clinical pictures, characterized by chronic hyperglycemia due to abnormal insulin secretion or action. Type 1 diabetes mellitus is the most common type of diabetes mellitus in children and adolescents, accounting for about 90% of diabetes in the population under the age of 18. The etiopathogenesis of type 1 diabetes is multifactorial. The disease occurs as a result of the interaction of three factors: genetic predisposition, environmental factors and the immune response. Research in recent years has focused on the involvement of Epstein–Barr virus (EBV) in the pathogenesis of type I diabetes. The goals of treating type 1 diabetes include maintaining blood-glucose, fructosamine and glycated hemoglobin (HbA1c) levels; therefore, the main purpose of this study was to evaluate the effect of EBV infection on the activation of selected immune cells, fructosamine levels and HbA1c levels in children with type I diabetes. Based on our study, we found a lower percentage of CD8+ T lymphocytes with expression of the CD69 molecule in patients with anti-VCA antibodies in the IgG class, and a lower percentage of CD8+ T lymphocytes with expression of the CD25+ molecule in patients with anti-EBNA-1 antibodies in the IgG class, which may indicate limited control of the immune system during EBV infection in patients. There was a lower percentage of CD3+CD4+ T lymphocytes secreting IL-4 in the study group, indicating that a deficiency in IL-4 production may be related to the development of type 1 diabetes. There was an increase in the percentage of CD4+CD3+IL-10 lymphocytes in the study group with anti-VCA antibodies present in the IgG class and anti-EBNA-1 antibodies in the IgG class compared to the patients without antibodies. In addition, there was a significant increase in fructosamine levels and higher glycated hemoglobin levels in the study group with antibodies to EBV antigens. In addition, an increase in the percentage of T lymphocytes with a CD4+CD3+IL-17+ phenotype in the patients with anti-VCA IgG antibodies was confirmed, and higher HbA1c levels may suggest that EBV infection is accompanied by an increase in IL-17 secretion.

## 1. Introduction

Type 1 diabetes is the most common type of diabetes in children and adolescents. It accounts for about 90% of diabetes cases in the population under the age of 18 [1]. A meta-analysis of data from around the world on the epidemiology of type 1 diabetes showed geographical differences The highest incidence rate of type 1 diabetes was recorded in Europe and North America and the lowest was recorded in Africa, South America and Asia. Diabetes is a wide group of metabolic diseases of different pathogeneses, etiologies and clinical pictures, characterized by chronic hyperglycemia as a result of incorrect insulin action [1,2,3]. Type 1 diabetes mellitus (DM1) is associated with incomplete or complete insulin deficiency, triggered by the destruction of pancreatic β cells [3]. It is divided into two types, i.e., diabetes mellitus type 1A (DM1A) and diabetes mellitus type 1B (DM1B) [1,4,5]. The literature is dominated by a complex model of etiopathogenesis, according to which the disease occurs as a result of the interaction between three factors: genetic predisposition, environmental factors and immune response [6,7]. Intensive research is currently underway on the microbiota as a potential factor contributing to the development of type 1 diabetes [8,9,10,11,12]. In addition, research studies indicate the involvement of viruses in the pathogenesis of fulminant type 1 diabetes [13]. This type of diabetes is characterized by a sudden onset, with marked hyperglycemia, ketoacidosis, normal glycated hemoglobin levels and the absence of antibodies against pancreatic islets [13,14,15]. According to the available research studies, cell-mediated immunity plays an important role in the development of autoimmunity, while the role of humoral immunity has not yet been sufficiently investigated [16,17,18,19]. In children and adolescents, the onset of type 1 diabetes is usually acute. The first symptoms of diabetes usually begin a few days or weeks before diagnosis. The characteristic symptoms of patients diagnosed with type 1 diabetes include: polydipsia, polyuria, involuntary urination and nocturia; some patients may experience weight loss despite an increased appetite [20,21]. Patients with type 1 diabetes require comprehensive treatment, including insulin therapy, physical activity, education (for their families, as well as for themselves), an appropriate diet and psychological support. The goals of the treatment of type 1 diabetes include: maintaining blood-glucose and fructosamine concentrations at values close to normal, maintaining the level of glycated hemoglobin (HbA1c, hemoglobin A1c) and balancing lipid metabolism [4,5]. The potential role of the Epstein–Barr virus (EBV) as a potential etiological agent of autoimmune diseases has been of great interest to many scientists in recent years [22,23]. Specific antibodies against EBV antigens are detected in approximately 95% of the human population [24,25]. After the virus enters the host cells, the infection can proceed in two ways, causing the autoimmunity process. In immunocompromised states, the virus may move from the latent phase to the lytic phase [26]. A significant phenomenon in the EBV virus, underlining its important role in the pathogenesis of autoimmune diseases, is the activation of autoreactive B lymphocytes [27,28,29,30], causing their proliferation and differentiation into effector cells, which occurs after contact with the antigen. The EBV is involved in the modification of the course of many autoimmune diseases, as evidenced by the presence of EBV genetic material in the organs of patients affected by the autoimmune process [31,32,33,34]. Studies indicate that this virus may also play an important role in the pathogenesis of type I diabetes. The aim of this study was to assess the impact of EBV infection on the activation of selected cells of the immune system, the concentration of fructosamine and the level of HbA1c in children with type I diabetes. For this purpose, we determined the percentage of T and B lymphocytes with the expression of selected activation markers (CD69+ and CD25+) and T lymphocytes (CD3+ and CD4+) with the intracellular expression of selected cytokines (IFN-γ, IL-2, IL-4, IL-10, IL-17) in a group of patients with type 1 diabetes and in a control group. In addition, the assessment of the concentration of antibodies against EBV antigens (anti-VCA in the IgM and IgG class and anti-EBNA-1 in the IgG class) was analyzed, as well as the level of glycated hemoglobin and the concentration of fructosamine in relation to selected indicators of EBV reactivation in patients from the research and control groups.

## 2. Results

### 2.1. Evaluation of Selected Subpopulations of Lymphocytes

The immunophenotype of the basic subpopulations of peripheral blood lymphocytes was assessed in all the patients from the study group and the control group. The conducted analysis showed no statistically significant differences in the percentage or absolute number of the main populations of lymphocytes in the study groups. A comparison of the absolute number and percentage of selected lymphocyte populations is presented in Table 1.

### 2.2. Evaluation of the Percentage of Lymphocytes Expressing Selected Activation Markers on T and B Lymphocytes

**Figure 1:**
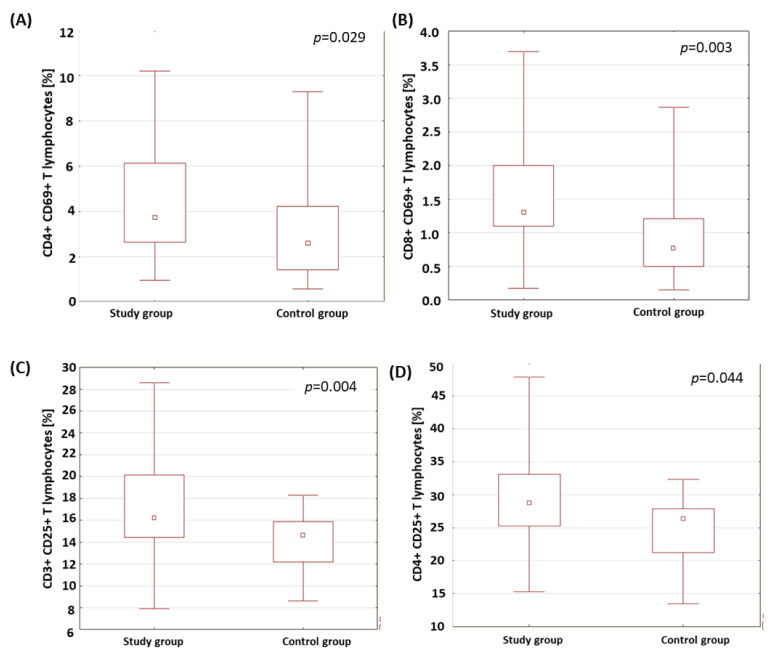
Comparison of the percentage of selected subpopulations of lymphocytes with CD69- and CD25-antigen expression in the study group and the control group. (**A**) Comparison of the percentage of CD4+ T lymphocytes with CD69-antigen expression in both analyzed groups; (**B**) comparison of the percentage of CD8+ T cells with CD69-antigen expression in both analyzed groups; (**C**) comparison of the percentage of CD3+ T cells with CD25-antigen expression in both analyzed groups; (**D**) comparison of the percentage of CD4+ T cells with CD25-antigen expression in both analyzed groups.

A statistically relevant higher percentage of CD4+ T lymphocytes expressing the CD69 marker was found in the group of patients with type 1 diabetes than in the control group (*p* = 0.029; Figure 1A). ‘The patients with type 1 diabetes were characterized by a statistically significantly higher percentage of CD8+ T lymphocytes expressing the CD69 molecule than the healthy subjects (*p* = 0.003; Figure 1B). The results obtained are presented in Table 2. The percentage of T and B lymphocytes expressing CD25 antigens was analyzed in the group of patients with type 1 diabetes and in the control group. The results obtained are presented in Table 2. The analysis showed a statistically relevant higher percentage of CD3+ T lymphocytes expressing the CD25 antigen in the group of patients with type 1 diabetes compared to the control group (*p* = 0.004; Figure 1C). The percentage of CD4+ T lymphocytes expressing the CD25 antigen in the patients with type 1 diabetes was statistically significantly higher than in the control group (*p* = 0.044; Figure 1D).

### 2.3. Evaluation of Lymphocytes with Intracellular Expression of Selected Cytokines

**Figure 2:**
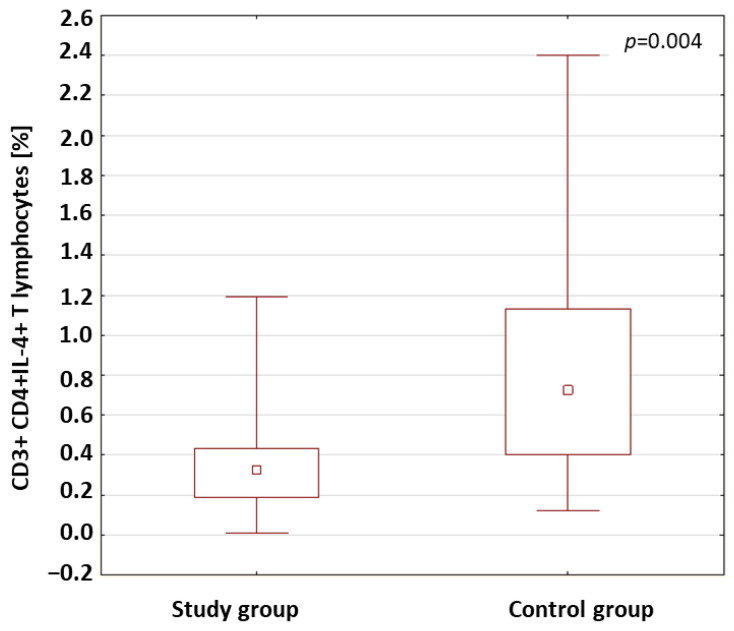
Comparison of the percentages of CD3+CD4+ T cells with intracellular expression of IL-4 in the study group and the control group.

The percentage of CD3+CD4+ T lymphocytes with the intracellular expression of IFN-γ+, IL-2, IL-4, IL-10 and IL-17 in the patients with type 1 diabetes and in the control group was analyzed. The results obtained are presented in Table 3. The analysis of the results showed that the percentage of CD3+CD4+ T cells with intracellular expression of IL-4 was significantly lower in the patients with type 1 diabetes compared to the percentage of these cells in the control group (*p* = 0.004; Figure 2).

### 2.4. Evaluation of Antibodies against EBV Antigens

**Figure 3:**
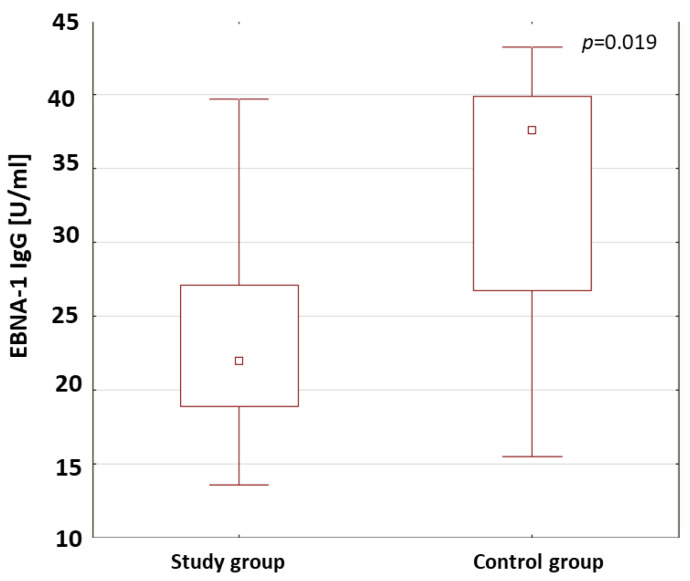
Comparison of the concentrations of anti-EBNA-1 antibodies in the IgG class in the study group and the control group.

The anti-VCA antibodies in the IgM and IgG class and the anti-EBNA-1 in the IgG class were assessed both in the patients with type 1 diabetes and in the control group. The results are shown in Table 4. The anti-VCA antibodies in the IgM class were detected in 48.5% of the patients with type 1 diabetes. The anti-VCA antibodies in the IgM class were not found in the control group. In the study group, the presence of anti-VCA antibodies in the IgG class was found in 69.8% of the patients and, in the control group, in 78.6%. The analysis of the results showed no statistically relevant differences in the concentrations of the anti-VCA antibodies in the IgG class in the study groups. The rates of the presence of anti-EBNA-1 antibodies in the IgG class in the patients were 48.8% in the study group and in 50% in the control group. In the patients with type 1 diabetes, the concentration of anti-EBNA-1 antibodies in the IgG class was statistically significantly lower compared to those in the control group (*p* = 0.019; Figure 3).

### 2.5. Evaluation of Selected Subpopulations of Lymphocytes in Patients with Type 1 Diabetes in Relation to the Presence of Antibodies against Antigens of the EBV Virus

The percentages CD3+ T lymphocytes, CD19+ B lymphocytes, CD3+CD4+ and CD3+CD8+ T lymphocytes and NK cells in the patients with type 1 diabetes were analyzed in relation to the presence of anti-VCA antibodies in the IgM class. There were no differences in the percentages or the absolute values of the selected subpopulations of lymphocytes in the patients with type 1 diabetes in relation to the presence of anti-VCA antibodies in the IgM class. The results are shown in Table 5.

**Figure 4:**
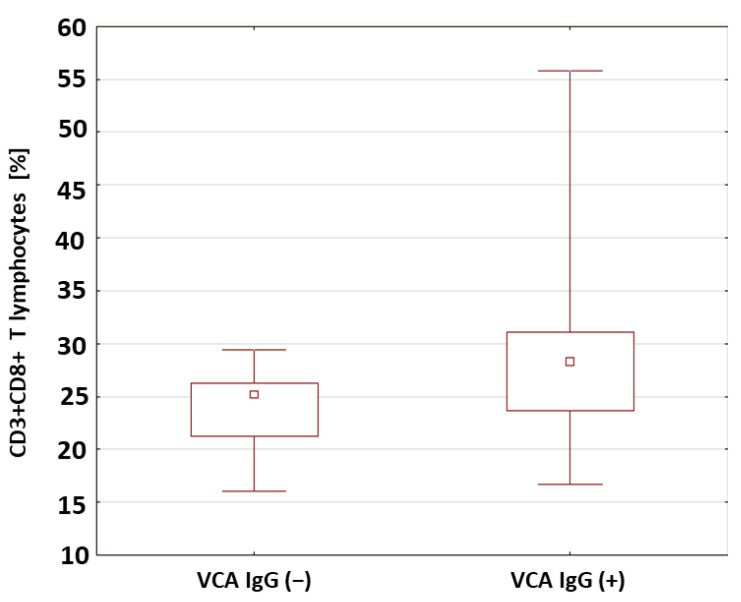
Comparison of the percentages of CD8+CD3+ T cells in a group of patients with type 1 diabetes in relation to the presence of anti-VCA antibodies in the IgG class.

In the patients with type 1 diabetes with anti-VCA IgG antibodies, A statistically relevant higher percentage (*p* = 0.033) (Figure 4) and absolute number (*p* = 0.041) of CD3+CD8+ T lymphocytes were found compared to the patients without anti-VCA antibodies. The percentages and absolute values of the main lymphocyte populations in the group of patients with type 1 diabetes were also analyzed in relation to the presence or absence of anti-EBNA-1 antibodies in the IgG class. Based on the obtained results presented in Table 5, there were no significant differences between the study groups.

### 2.6. Evaluation of the Percentage of Lymphocytes Expressing Selected Activation Markers in Patients with Type 1 Diabetesin Relation to the Presence of Antibodies against EBV Antigens

**Figure 5:**
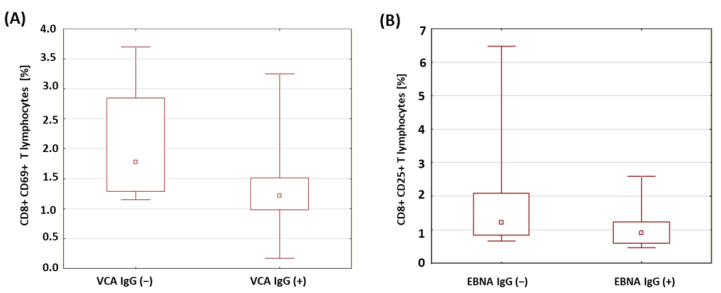
Comparison of the percentages of selected subpopulations of lymphocytes expressing the CD69 antigen in a group of patients with type 1 diabetes in relation to the presence of anti-VCA and anti-EBNA antibodies in the IgG class. (**A**) Comparison of the percentages of CD8+ T cells with the expression of the CD69 antigen in a group of patients with type 1 diabetes in relation to the presence of anti-VCA antibodies in the IgG class; (**B**) comparison of the percentages of CD8+ T cells with the expression of the CD25 antigen in a group of patients with type 1 diabetes in relation to the presence of anti-EBNA-1 antibodies in the IgG class.

The percentages of T and B lymphocytes expressing CD69 antigens in a group of patients with type 1 diabetes was analyzed in relation to the presence of anti-VCA antibodies in the IgM and IgG classes. The results obtained are presented in Table 6. There were no significant differences in the percentages of the T and B lymphocytes expressing the CD69 antigen in the patients with type 1 diabetes in relation to the presence of anti-VCA antibodies in the IgM class. However, it was shown that the percentage of CD8+CD69+ T cells was significantly lower in the group of patients with anti-VCA antibodies in the IgG class (*p* = 0.029; Figure 5A). Moreover, based on the conducted analysis, no statistically significant differences were found in the percentage of T and B lymphocytes expressing the CD69 antigen in the patients with type 1 diabetes in relation to the presence of anti-EBNA-1 antibodies in the IgG class.

A similar analysis was performed for the expression of the CD25 antigen on the T and B lymphocytes in the patients with type 1 diabetes in relation to the presence of antibodies against EBV antigens. The obtained results are presented in Table 7. There were no statistically relevant differences between the percentages of T and B lymphocytes expressing the CD25 antigen in the patients with type 1 diabetes in relation to the presence of anti-VCA antibodies in the IgM and IgG class. Only a significantly lower percentage of CD8+ T lymphocytes with the expression of the CD25 antigen was found in the group of patients with type 1 diabetes with anti-EBNA-1 antibodies in the IgG class compared to the group of patients with type 1 diabetes without anti-EBNA-1 antibodies in the IgG class (*p* = 0.042; Figure 5B).

### 2.7. Evaluation of the Percentage of Lymphocytes with Intracellular Expression of Selected Cytokines in Patients with Type 1 Diabetes in Relation to the Presence of Antibodies against Antigens of the EBV Virus

The results of a comparative analysis of the percentages of CD3+CD4+ T lymphocytes with intracellular expression of the cytokines IFN-γ, IL-2, IL-4, IL-10 and IL-17 in the patients with type 1 diabetes with anti-VCA antibodies in the IgM class and in the patients without detected anti-VCA antibodies in the IgM, IgG and anti-EBNA-1 in the IgG class are presented in Table 7.

**Figure 6:**
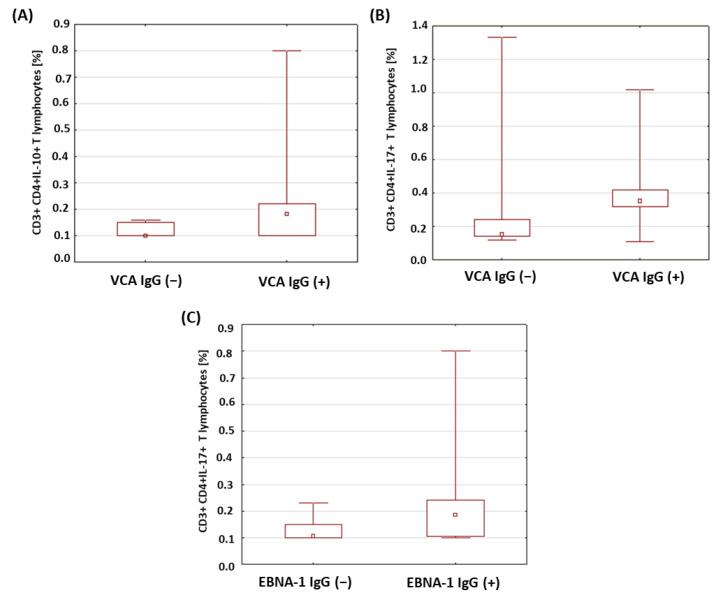
Comparison of the percentages of selected subpopulations of lymphocytes showing intracellular expression of the studied interleukins in patients with type 1 diabetes in relation to the presence of anti-VCA and anti-EBNA-1 antibodies in the IgG class. (**A**) Comparison of the percentage of CD4+CD3+ T cells with intracellular IL-10 expression in type 1 diabetic subjects according to the presence of anti-VCA IgG antibodies; (**B**) comparison of the percentage of CD4+CD3+ T cells with intracellular expression of IL-17 in type 1 diabetic subjects according to the presence of anti-VCA IgG antibodies; (**C**) comparison of the percentage of CD4+CD3+ T cells with intracellular IL-10 expression in type 1 diabetic subjects according to the presence of anti-EBNA-1 IgG antibodies.

Based on the conducted analyses, no relevant differences were found in the percentage of CD3+CD4+ lymphocytes with intracellular expression of the selected cytokines in the patients with type 1 diabetes in relation to the presence of anti-VCA antibodies in the IgG class. Only a statistically significantly higher percentage of CD3+CD4+IL-10+ T cells (*p* = 0.011; Figure 6A) and CD3+CD4+IL-17+ T cells (*p* = 0.030; Figure 6B) was found among the patients with the presence of anti-VCA antibodies in the IgG class. In addition, we showed that in the group of patients with type 1 diabetes with anti-EBNA-1 antibodies in the IgG class, the percentage of CD3+CD4+IL-10+ T cells was significantly higher (*p* = 0.033; Figure 6C).

### 2.8. Evaluation of Selected Parameters of Carbohydrate Balance in Patients with Type 1 Diabetes in Relation to the Presence of Antibodies against Antigens of the EBV Virus

**Figure 7:**
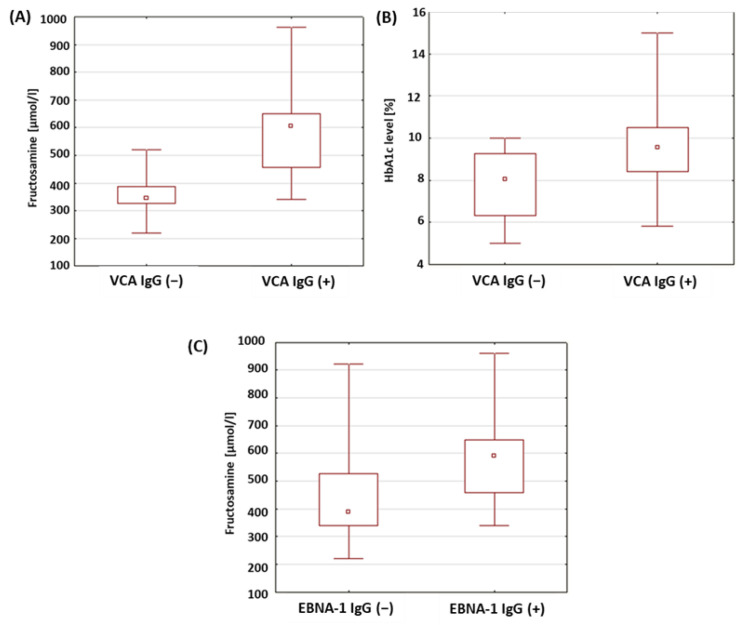
Evaluation of selected parameters of carbohydrate balance in patients with type 1 diabetes in relation to the presence of antibodies against antigens of the EBV virus. (**A**) Comparison of fructosamine concentrations in a group of patients with type 1 diabetes in relation to the presence of anti-VCA antibodies in the IgG class; (**B**) comparison of the levels of HbA1c in a group of patients with type 1 diabetes in relation to the presence of anti-VCA antibodies in the IgG class; (**C**) comparison of fructosamine concentrations in a group of patients with type 1 diabetes in relation to the presence of anti-EBNA-1 antibodies in the IgG class.

The levels of glycosylated hemoglobin and the fructosamine concentrations in a group of patients with type 1 diabetes was analyzed in relation to the presence of anti-VCA antibodies in the IgM, IgG and anti-EBNA-1 in the IgG class. The results obtained are presented in Table 8. There were no statistically significant differences in the levels of HbA1c and fructosamine between the study groups in relation to the anti-VCA antibodies in the IgG class. In the type 1 diabetic patients with anti-VCA IgG antibodies, significantly higher fructosamine concentrations were found compared to the patients without anti-VCA IgG antibodies (*p* < 0.001; Figure 7A). A statistically relevant higher level of glycated hemoglobin was also found in the group of patients with type 1 diabetes with anti-VCA antibodies in the IgG class (*p* = 0.017; Figure 7B). Moreover, a significantly higher concentration of fructosamine was found in the group of patients with type 1 diabetes with anti-EBNA-1 IgG antibodies in relation to the group of patients with type 1 diabetes without anti-EBNA-1 antibodies in the IgG class (*p* = 0.015; Figure 7C).

## 3. Discussion

The complex mechanism of the development of type 1 diabetes makes it difficult to study the etiopathogenesis of the disease. For example, the analysis of the share of viruses is a very serious challenge, requiring many years of observation in different populations and age groups. Research is underway around the world to determine which specific viral infections are associated with type 1 diabetes. An analysis of the incidence of infections in children with type 1 diabetes showed an increase in the incidence of infections in the autumn and winter seasons, which probably contributes to the development of the disease [35,36,37]. Immune mechanisms or certain microbes appear to be involved in the destruction of insulin-producing pancreatic cells [38,39]. The homology of the viral antigens with the antigens of the body’s own cells is a possible cause of the immune response that is directed against the self-antigens [40,41,42,43,44]. Rasmussen et al. showed that in children with a genetic predisposition towards the development of diabetes, the diagnosis of this disease was much more likely in newborns who had a viral infection of the lower respiratory tract [42]. It is suggested that some of the lymphocytes activated during viral infection with one pathogen may cross-recognize other viruses. In this way, memory T cells may accumulate with a specificity unrelated to the original stimulator. Accumulated in this way, memory lymphocytes respond much more rapidly and effectively to antigens, which may lead to the development of autoimmune diseases in people with a genetic predisposition to these diseases [45].

The confirmation of the immune response to the presence of antigens is the presence of activation markers on the cell surface. The early stage of the lymphocyte response includes the detection of CD69-antigen expression, which occurs 2–4 h after the first stimulatory signals appear [46,47,48]. The late stage of activation is characterized by the presence of the CD25 antigen on the cell surface, the expression of which persists after the elimination of the stimulating factor [49]. Scientists found a higher expression of CD69 and CD25 antigens in patients with type 1 diabetes than in a control group [50,51]. In our own research, a higher percentage of CD8+ T lymphocytes expressing the early activation markers, CD69 (*p* = 0.003), CD4+ CD69+ T lymphocytes (*p* = 0.029), as well as the CD3+ T lymphocytes expressing the late activation marker, CD25 (*p* = 0.004), along with the CD4+CD25+ phenotype (p = 0.044) in patients with diabetes than in a control group. The obtained results are consistent with the reports by Giordano et al. [52] and indicate that the lymphocytes of diabetic patients respond to antigen stimulation; the observation of a higher percentage of these in the diabetic patients than in the control group indicates a much higher exposure to antigens.

The mechanism by which viruses contribute to the destruction of pancreatic beta cells remains unknown. Infection can directly lead to the lysis of pancreatic cells. The exposure of altered pancreatic beta-cell antigens to cells of the immune system and the increased expression of major histocompatibility complex (MHC) molecules may be among the factors contributing to the development of type 1 diabetes [40,41]. The activation of antigen-presenting cells (APC) and the subsequent inflammatory reaction does not always cause the development of an autoimmune disease; therefore, it seems that viruses may not be responsible for the initiation of autoimmune processes, but increase their probability [45,53,54]. Viruses, as superantigens, contribute to the activation and proliferation of specific lymphocytes. If autoreactive lymphocytes recognize superantigens, then viruses may be involved in the development of autoimmune diseases [55].

Another mechanism responsible for the development of the disease is the induction of pro-inflammatory cytokines in the environment of the pancreas [56,57]. The time at which they are secreted is of great importance in the development of the disease. The production of TNF in the early stage of type 1 diabetes contributed to its progression, while the appearance of cytokines in the later stage of the disease was associated with a decrease in the number of autoreactive lymphocytes [58,59]. Inflammatory cytokines and chemokines may be key mediators controlling the movement of virus-activated autoreactive T cells, which enables the infiltration of pancreatic islets by these cells [45]. Knowledge about the relationship between viral infections and the development of type 1 diabetes is limited. Research conducted by Łuczyński et al., Szypowska et al. and Zahran et al. showed that in children with newly diagnosed diabetes, the percentage of regulatory T lymphocytes (Treg) is reduced [60,61,62]. Poor glycemic control and, thus, hyperglycemia, may contribute to a reduction in the percentage of Treg lymphocytes [63]. Viral infections may be factors in the disturbance of the balance between autoreactive lymphocytes and Treg lymphocytes [40]. Experimental depletion or genetic Treg deficiency contributes to the progression of type 1 diabetes [64]. The balance between Treg cells and effector cells is maintained by IL-2. Abnormalities in the structure of its receptor have been observed in type 1 diabetes and the disorder of IL-2 synthesis by effector T cells has been suggested as a factor in the pathogenesis of the disease [65,66,67]. The administration of antibodies binding the IL-2 receptor increased the incidence of the disease in mice. One of the effects of IL-2 is the activation of Tregs. Deficiency in IL-2, which is the activator of Tregs, explains the dysfunction in diabetes [68,69]. Lindley et al. showed a significantly higher percentage of CD4+ T lymphocytes expressing CD25 accompanied by CD69 expression in diabetic patients. Recently activated effector cells are characterized by CD69 [70]. Zahran et al. found a relationship between the percentage of T cells with the CD4+CD25+ phenotype and the concentration of C-peptide in children with type 1 diabetes [62]. However, El-Masry et al. did not confirm the relationship between HbA1c and the percentage of this population [71]. Repeated exposure to antigens may protect against the development of type 1 diabetes. The observed association of repeated infections with an increase in the percentage of Treg lymphocytes that have beneficial properties in type 1 diabetes or the functionally depleted lymphocytes formed after infection may explain the positive effect of exposure to antigens [45]. Studies by Cinek et al. and Frederiksen et al. showed that enterovirus infections are detected much more often in children with an active autoimmune response towards pancreatic islets than in healthy children [72,73]. It seems that cytomegalovirus and EBV virus may also play an important role in the pathogenesis of type 1 diabetes among herpesviruses. Currently, the mechanisms by which these viruses may contribute to the development of the disease are not known [35].

The EBV virus persists in the body, with a periodic ability to reactivate [35]. Chen et al. demonstrated the presence of EBV in the pancreas [74]. The reactivation of the virus in immunocompromised individuals may be associated with a diagnosis of type 1 diabetes [75]. Studies by Hyota et al. showed the presence of antibodies against the EBV capsid and early antigens in newly diagnosed children with diabetes. It was also found that the level of antibodies against the EBV capsid was much lower than in a group of healthy children. The authors interpreted this result as an abnormality in the EBV-specific immune response [76]. Similarly, in the study of the dissertation, a significantly lower concentration of anti-EBNA-1 IgG antibodies (*p* = 0.019) was found in diabetic patients than in a control group. The obtained results suggest that EBV infection may accompany the development of type 1 diabetes. Our own research showed a significantly lower percentage of CD8+ T lymphocytes expressing the CD69 antigen in patients with anti-VCA antibodies in the IgG class (*p* = 0.029). Draborg et al. evaluated the effect of EBNA-1-peptide stimulation on CD69 expression in patients with systemic lupus erythematosus (SLE). A significant reduction in CD69-antigen expression was demonstrated [77]. In diabetic patients with anti-EBNA-1 antibodies in the IgG class, a decrease in the percentage of CD8+ T lymphocytes expressing the CD25 antigen was also observed (*p* = 0.042). Draborg et al. [77] suggest that the presence of lower numbers of activated EBV-specific lymphocytes is associated with poor control of EBV infection. Frequent, recurring infections can cause a strong activation of lymphocytes, which then become depleted. The decrease in reactivity of EBV-specific lymphocytes may be a consequence of a limited or defective immune response.

Kang et al. [78] observed a decrease in the percentage of CD8+ T cells producing IFN-γ and IL-2 and an increase in CD8+ T cells producing IL-4 and IL-10 in patients with SLE. The patients showed a defective response of CD8+ T lymphocytes, which led to a lack of control of EBV latency, which was observed through the detection of EBV DNA in patients. An increase in the number of EBV-specific CD8+ T lymphocytes was observed with a simultaneous weakened response to stimulation with EBV antigens. Kang et al. [78] suggest that the detection of EBV DNA with increasing numbers of EBV-specific CD8+ T cells indicates an insufficient EBV-specific response. In patients with SLE, the lack of mechanisms to keep the virus in latency has been found to be a likely cause of viral reactivation.

The reduction in the number of EBV-specific lymphocytes contributed to the increase in the severity of the disease [77]. In our own research, a significantly higher number of CD3+CD8+ T lymphocytes (*p* = 0.041) was found in patients with anti-VCA IgG antibodies. It was also found that among the population of CD8+ T lymphocytes, there was a significantly lower percentage of lymphocytes expressing the CD69 antigen in this group of patients. Draborg et al. [77] emphasize that the simultaneous observation of a reduced concentration of anti-EBNA-1 antibodies and a weakened specific EBV response may result in the transformation of the immune response from cellular to humoral. The authors believe that this is an attempt to control the possible reactivation of the infection. In the lytic phase of EBV infection, infected cells undergo apoptosis and release viral antigens. The lack of control of infection by cellular mechanisms contributes to the production of antibodies towards the released antigens [77]. Furthermore, EBV-specific CD8+ T cells produce IL-4, IL-10 and IL-13, which are involved in B-cell activation and proliferation. One hypothesis assumes that after primary infection with EBV in individuals with a genetic predisposition towards the development of autoimmune diseases, the virus-infected activated autoreactive B lymphocytes accumulate in the organs in which the target antigens are located [79,80]. The expression of the viral LMP protein protects B lymphocytes from apoptosis [81,82].

The EBV virus encodes a human IL-10 homolog. In the presence of viral IL-10, the lymphocyte-effector response can be converted to a Th2-mediated response associated with IL-10 production [83]. Marshall et al. showed that the EBV LMP1 protein stimulates the production of IL-10 by CD4+ T cells. The LMP1 protein inhibits the proliferation and release of IFN-γ by mitogen-stimulated mononuclear cells. Lymphocyte exposure to the LMP1 protein leads to the transformation of the Th1-mediated response into a regulatory response related to the production of IL-10 [83].

Our own research showed a significantly higher percentage of IL-10 secreting CD4+CD3+ T lymphocytes in a group of patients with anti-VCA antibodies in the IgG class than in patients without antibodies (*p* = 0.011). Similarly, a significantly higher percentage of these lymphocytes was found in patients with anti-EBNA-1 IgG antibodies than in those without antibodies (*p* = 0.033). The finding of a significantly higher percentage of CD4+CD3+ T lymphocytes secreting IL-10 in diabetic patients with anti-VCA and anti-EBNA-1 antibodies in the IgG class proves the transformation of the immune response into a humoral response. The increase in the number of IL-10-producing lymphocytes may be an attempt to activate one of the regulatory mechanisms that may keep the virus in a latent state.

The induction of a Th1-mediated response in diabetic patients may be related to the activation of TLRs after binding the appropriate ligand [84]. Al Shamsi et al. conducted a study in which mice lacking IFN-γ were shown to be unable to develop diabetes when stimulated with TLR2 ligands [85]. In patients with type 1 diabetes, Zhang et al. found higher concentrations of IFN-γ and IL-2 compared to the control group [86]. Abdel-Latif et al. showed significantly higher levels of IL-10 and IL-17 in patients with type 1 diabetes during enterovirus infection than in patients without infection. The immune response to viral infection included an increase in IFN-γ and IL-17 and a decrease in IL-4 and IL-13. The production of IFN-γ and IL-17 exacerbated the disease [87]. In addition, IL-17 plays an important role in patients with type 1 diabetes. The cytokine impairs glucose and insulin metabolism in young diabetic mice and increases the production of IL-6, which induces insulin resistance [88]. Our own research showed a significantly higher percentage of CD4+CD3+ T helper lymphocytes secreting IL-17 in patients with anti-VCA antibodies in the IgG class (*p* = 0.030). Obtaining significantly higher concentrations of fructosamine and HbA1c and the percentage of lymphocytes with the CD4+CD3+IL-17+ phenotype suggests that impaired glucose metabolism in patients with type 1 diabetes with anti-VCA antibodies is accompanied by an increase in the production of IL-17 by helper T cells.

Our own studies showed that in a group of patients with anti-VCA antibodies in the IgG class, an increase in the percentage of CD4+CD3+IL-10 lymphocytes (*p* = 0.011) and the concentration of fructosamine (*p* < 0.001) and HbA1c (*p* = 0.017). In the group of patients with the presence of anti-EBNA-1 antibodies in the IgG class, a significantly higher concentration of fructosamine (*p* = 0.015) was found than in patients without the presence of these antibodies. The results of the study suggest that the increase in IL-10 production by helper T lymphocytes may not be related to impaired glucose metabolism in this group of patients. On the other hand, this effect appears to balance the occurrence of the active phase of EBV infection. The increase in IL-10 production by T helper cells may be a latent mechanism for the virus. Accompanying disturbances in the parameters of carbohydrate balance may result from the stimulation of the immune system by viral antigens or occur independently of infection, as a consequence of the resulting disturbances in the functioning of the immune system.

The anti-inflammatory cytokine, IL-4, exerts a cytoprotective effect on pancreatic cells [89]. It inhibits the production of cytokines that induce insulin resistance, such as tumor-necrosis factor or IL-6 [90]. Kamiński et al. found the role of IL-4 in regulating the synthesis of the nitric oxides contributing to the increase in the production of pro-inflammatory cytokines that damage the pancreas [91]. Chang et al. found that IL-4 increased cellular-glucose utilization and improved insulin action [92]. Studies by Müller et al. showed that the reduction in IL-4 concentrations may be a much stronger factor inducing the development of the disease than the action of IFN-γ [93]. In our own research, a significantly lower percentage of CD4+CD3+ T lymphocytes secreting IL-4 was found in the diabetic patients than in the control group (*p* = 0.004). The results of this study may support the hypothesis that a deficiency in IL-4 production by lymphocytes characterizes patients with type 1 diabetes and may contribute to the development of the disease.

## 4. Materials and Methods

### 4.1. Characteristics of Patients from the Research and Control Groups

This study included 43 children diagnosed with type 1 diabetes, hospitalized in the Department of Pediatric Endocrinology and Diabetology of the Medical University of Lublin between June 2015 and January 2016. People diagnosed with type 1 diabetes in accordance with the criteria of the Polish Diabetology Society qualified for the study. Criteria for inclusion in the study group: Obtaining informed, written consent for the examination of a parent or legal representative and, in addition, in the case of patients over 16 years of age, the consent of the child; diagnosis of type 1 diabetes. Criteria for exclusion from the study group: Acute infectious disease; severe systemic disease; concurrent autoimmune disease; use of medications affecting the immune system in the 3 months before the examination; transfusion of blood and blood products within 3 months before the examination. There were 21 girls (48.8%) and 22 boys (51.2%) in the study group. The ages of the children at the time of the examination ranged from 3.9 to 18 years, with an average of 13.58 years. The control group consisted of 20 children; 8 girls (40%) and 12 boys (60%). In these patients, there were no disorders of carbohydrate metabolism, autoimmune diseases or hypopituitarism. The participants in the study were aged from 7.75 to 14.4 years, with an average of 11.02 years. The characteristics of the study and control groups are presented in Table 9.

### 4.2. Material for Research

The material for the study was whole blood collected from the basilic vein into two 4.5-milliliter tubes containing disodium edetate. The collected material was used to assess the immunophenotypes of cells, including activation markers (CD69+ and CD25+), using flow cytometry. The intracellular expression of cytokines (IFN-γ+, IL-2, IL-4, IL-10 and IL-17) and the concentration of anti-VCA IgM, anti-VCA IgG and anti-EBNA-1 antibodies were assessed in the IgG class in the blood plasma using the ELISA technique. Glycated hemoglobin (HbA1c), fructosamine and blood counts were determined according to standard hospital-laboratory procedures (ALAB Medical Analyzes Laboratory). The obtained results were related to the reference values used in the laboratory.

### 4.3. Evaluation of the Subpopulation of Peripheral-Blood Lymphocytes by Flow Cytometry

**Figure 8:**
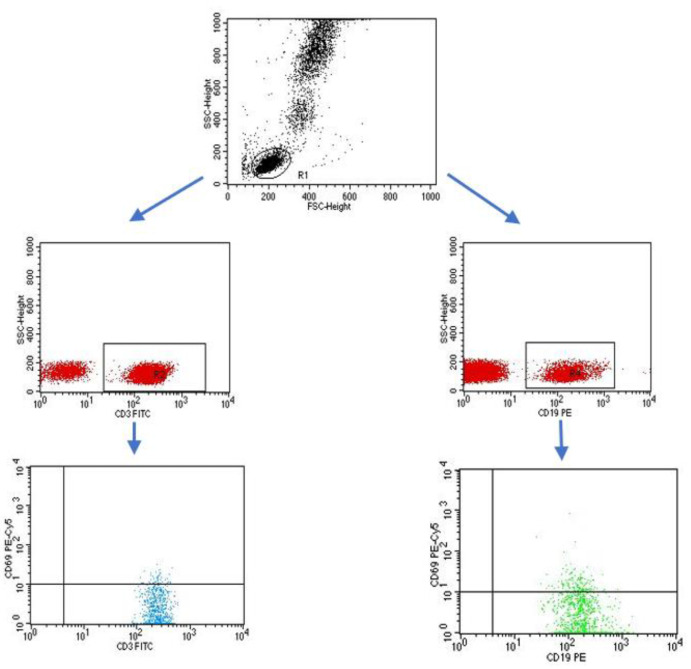
Sample analysis of the percentage of lymphocytes with the CD3+CD69+ and CD19+CD69+ phenotype.

**Figure 9:**
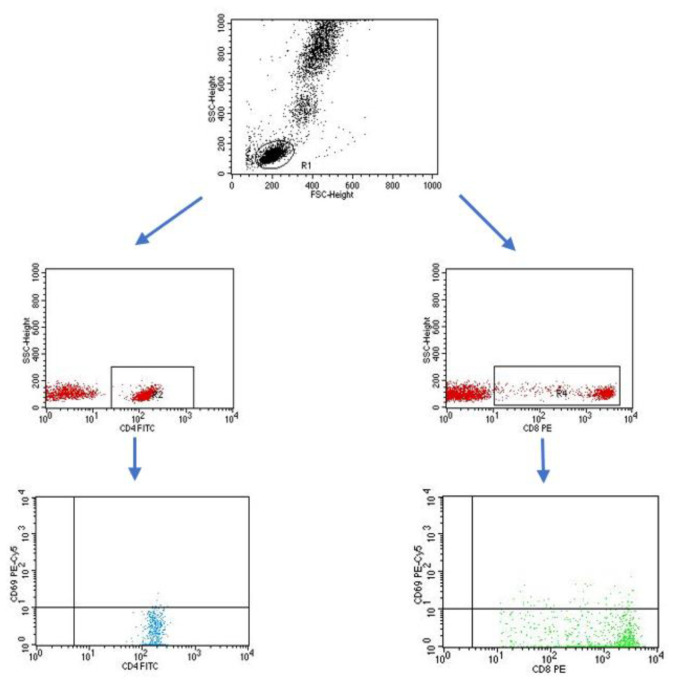
Sample analysis of the percentage of T cells with the CD4+CD69+ and CD8+CD69+ phenotype.

**Figure 10:**
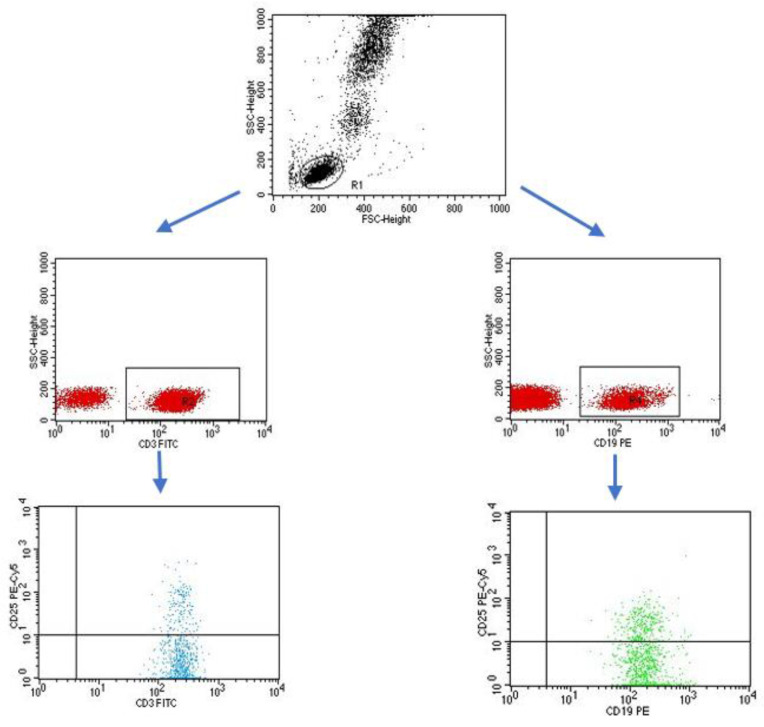
Sample analysis of the percentage of lymphocytes with the CD3+CD25+ and CD19+CD25+ phenotype.

A flow cytometer, FACSCalibur (Becton Dickinson, San Jose, CA USA), equipped with an argon laser with a wavelength of 488 nm was used to assess the immunophenotype of peripheral blood cells. The CellQuest computer program (Becton Dickinson, San Jose, CA, USA) was used to analyze the examined parameters. A CaliBRITE calibration kit (Becton Dickinson, San Jose, CA, USA) was used to optimize the flow-cytometer settings. In total, 20,000 cells were acquired for each of the test samples using a plotted lymphocyte gate (R1 region). The gate was created in the system of FSC and SSC parameters. Cell -distribution analysis, based on labeling of cells with antibodies directed to CD45 and CD14 antigens, allowed the purity of the gate to be determined. The percentage of cells positively labeled with monoclonal antibodies was evaluated. Whole blood was used to evaluate surface antigens. Appropriate monoclonal antibodies were added in an amount of 20 µL per tube. A total of 50 μL of whole blood was added to each of the tubes containing the monoclonal antibodies. This mixture was incubated for 20 min at room temperature. After incubation, 2 mL of lysis solution was added to each tube, followed by an 8-minute incubation at room temperature. In the next step, the cells were rinsed twice with phosphate-buffered saline (PBS) (700× *g*, 5 min) and immediately analyzed in a FacsCalibur flow cytometer (Becton Dickinson, San Jose, CA, USA). Sample cytometric analyses performed on patients with type 1 diabetes are presented in Figure 8, Figure 9 and Figure 10.

### 4.4. Isolation of Peripheral-Blood Mononuclear Cells

Mononuclear cells (PBMC, peripheral mononuclear blood cells) were isolated from the obtained peripheral blood. For this purpose, peripheral blood was diluted with 0.9% buffered saline (PBS) without calcium (Ca^2+^) and magnesium (Mg^2+^) salts (Biochrom AG, Berlin, Germany) in a ratio of 1:1. Next, the diluted blood was layered on 3 mL of Gradisol L (Aqua Medical, Pomorskie, Poland) with a specific gravity of 1.077 g/mL and centrifuged in a density gradient for 20 min at 700× *g*. The resulting mononuclear cell fraction was collected with Pasteur pipettes and washed twice for 5 min in PBS without Ca^2+^ or Mg^2+^ ions. The washed cells were then resuspended in 1 ml PBS without Ca^2+^ or Mg^2+^ ions and their number in a Neubauer chamber and viability were determined using trypan blue (0.4% Trypan Blue Soulution, Sigma, Schnelldorf, Germany). Viability below 90% was used as a criterion for disqualifying cells from further research. The PBMC was used to assess the percentage of helper T cells secreting cytokines (IFN-γ, IL-2, IL-4, IL-10, IL-17).

### 4.5. Cytokine Labeling

**Figure 11:**
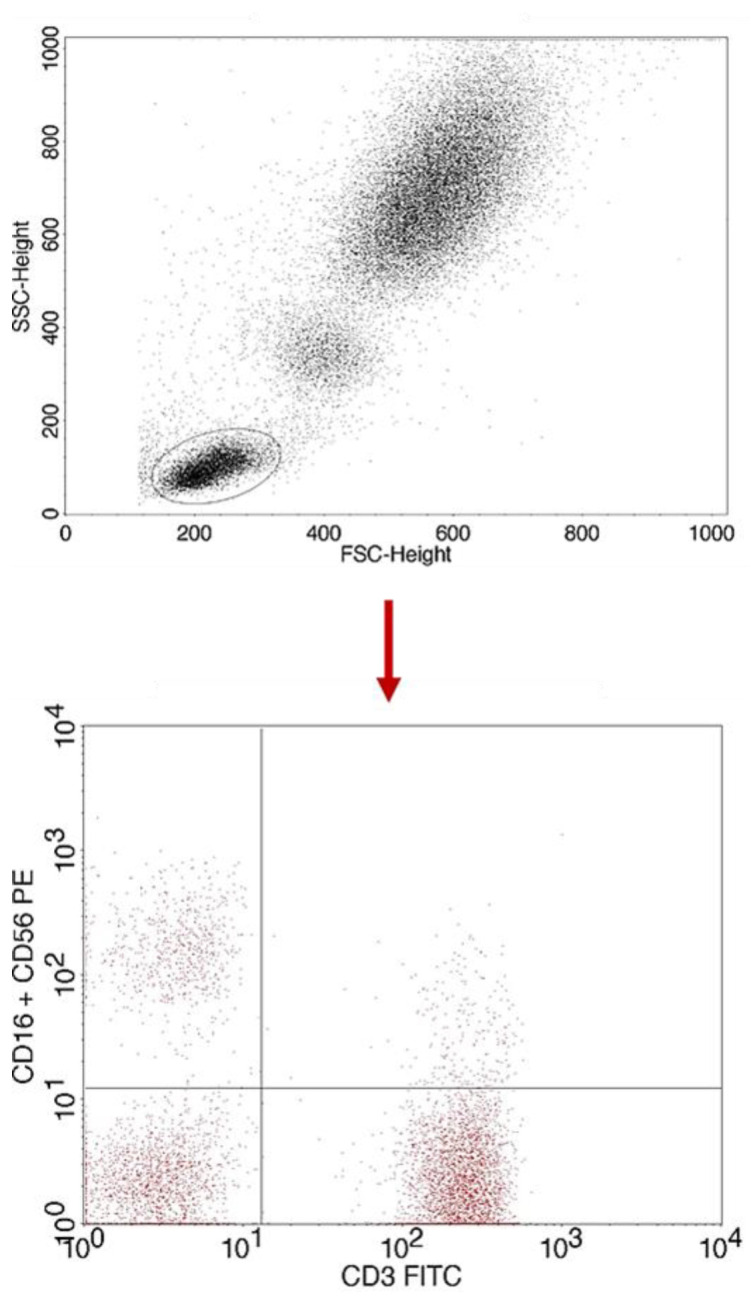
Sample analysis of the percentage of NK cells with the CD3-CD16+CD56 phenotype.

**Figure 12:**
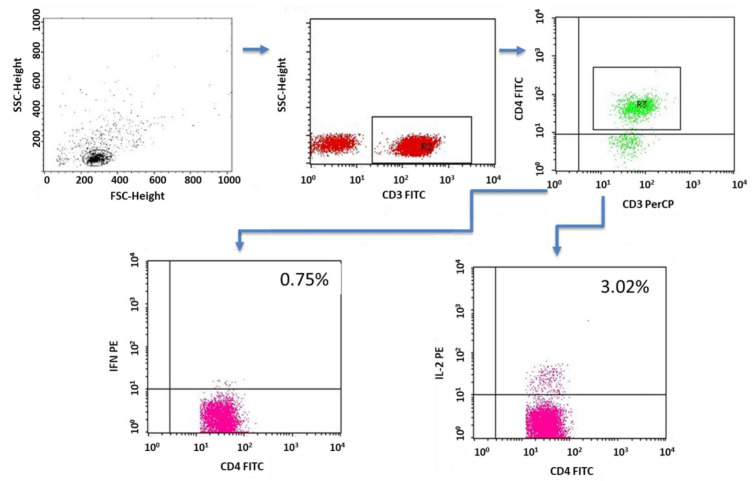
Sample analysis of the percentage of T lymphocytes with the CD4+CD3+IFNγ+ and CD4+CD3+IL-2+ phenotypes.

**Figure 13:**
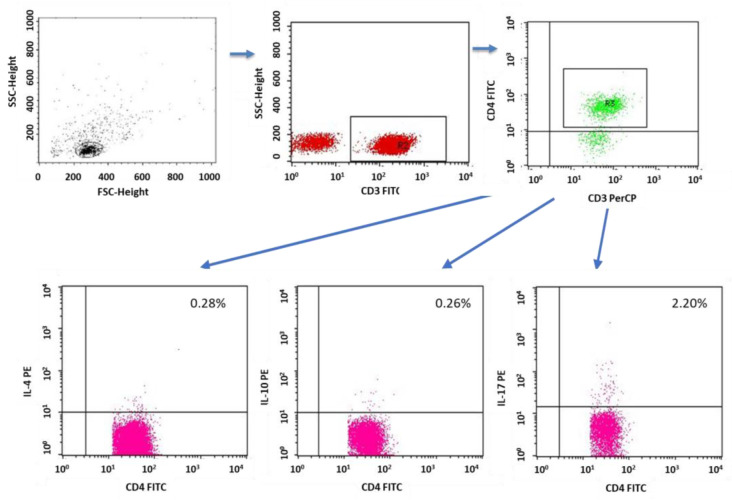
Sample analysis of the percentage of T cells with the CD4+CD3+IL-4+, CD8+CD3+IL-10+ and CD4+CD3+IL-17+ phenotypes.

**Figure 14:**
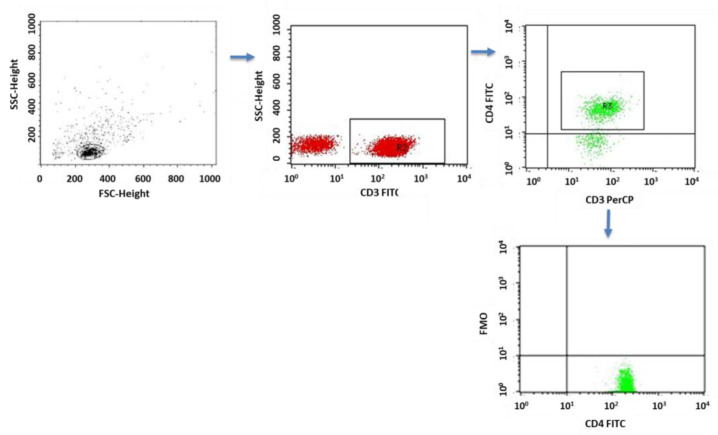
FMO control for cytometric analyses of cytokines.

**Figure 15:**
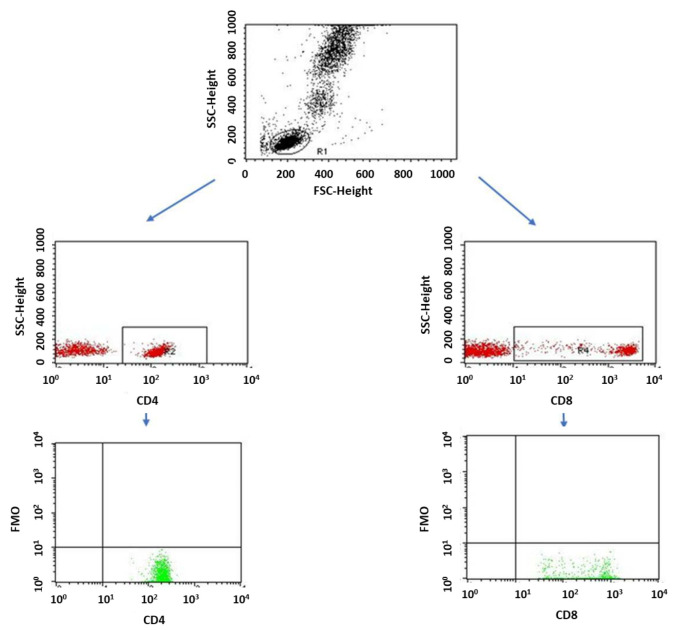
FMO control for cytometric analyses of CD69+ and CD25+ cells.

The PBMC incubations were performed in four-well plates (Nunc, Werder (Havel), Germany) for 4 h at 37 °C and 5% CO_2_ PBMCs (2 × 10^6^ cells/mL medium) suspended in medium containing RPMI 1640 (PanBiotech, Bayern, Germany) together with 2% human albumin (Baxter, Deerfield, IL, USA) and the following antibiotics: penicillin (100 IU/mL), streptomycin (50 µg/mL) and neomycin (100 µg/mL) (Sigma Aldrich, Schnelldorf, Germany). In addition to the medium, the following lymphocyte stimulators were added to the wells: PMA (phorbol myristate acetate, 50 ng/mL; and ionomycin, 1 µg/mL (Sigma Aldrich, Schnelldorf, Germany). At the same time, together with the stimulators, a protein-transport inhibitor, brefeldin A (Sigma Aldrich, Schnelldorf, Germany), was added in the amount of 10 µg/mL in order to accumulate the cytokines produced in the cell. After 4 h of incubation, the cells were labeled with antibodies directed against lymphocyte surface antigens (CD3, CD4). The samples were then fixed and permeabilized using the BD Cytofix/Cytoperm™ reagent kit (Becton Dickinson, ND, USA) according to the manufacturer’s protocol. To assess cytokine production, cells were incubated with 20 µL of the appropriate antibodies. Sample cytometric analyses performed on patients with type 1 diabetes are presented in Figure 11, Figure 12, Figure 13, Figure 14 and Figure 15.

### 4.6. Evaluation of the Concentrations of Antibodies against Epstein–Barr Virus Antigens in the Serum

Antibody concentrations were assessed by ELISA. The following kits were used for the determinations: anti-VCA IgM (Demeditec Diagnostics GmbH, Kiel, Germany), sensitivity 10 U/mL; anti-VCA IgG (Demeditec Diagnostics GmbH, Kiel, Germany), sensitivity 10 U/mL; and anti-EBNA-1 IgG (Demeditec Diagnostics GmbH, Kiel, Germany), sensitivity 10 U/mL. The VICTOR automatic reader (Perkin Elmer, Waltham, MA, USA) was used to read the light absorbance of the tested samples. Based on the standard curve, comprising standards with different concentrations and plotted by the WorkOut computer program, the concentrations of antibodies in the samples were assessed.

### 4.7. Statistical Analysis

The obtained research results were used to create a computer database and then subjected to statistical analysis using the Statistica 12 PL computer program (StatSoft, TIBCO Software Inc, Palo Alto, CA, USA). The level of statistical significance was *p* < 0.05. The assessment of the normality of the distribution of continuous variables was carried out using the Shapiro–Wilk test. The values of the analyzed continuous variables were presented in the form of medians, arithmetic means and standard deviations (SD), as well as extreme values (minimum and maximum). Between-group comparisons of independent variables were made using the Mann–Whitney U test.

## 5. Conclusions

Based on the study, we found a lower percentage of CD8+ T cells expressing the CD69 molecule in patients with anti-VCA IgG antibodies and a lower percentage of CD8+ T cells expressing the CD25+ molecule in patients with anti-EBNA-1 IgG antibodies, which may indicate the limited control of the immune system in the course of EBV infection in these patients. In addition, we showed that the lower percentage of CD3+CD4+ T cells secreting IL-4 in patients with type 1 diabetes supports the idea that a deficiency in IL-4 production may be associated with the development of type 1 diabetes. The finding of an increase in the percentage of CD4+CD3+IL-10 lymphocytes in the group of patients with anti-VCA IgG antibodies and anti-EBNA-1 IgG antibodies compared to the patients with no antibodies suggests that the increase in the production of IL 10 by helper T cells plays a role in balancing the production of pro-inflammatory cytokines and may be a latency mechanism. Moreover, the higher concentrations of fructosamine and levels of glycosylated hemoglobin in the group of patients with the presence of antibodies against antigens of the EBV virus, demonstrated in this study, suggest that one of the factors contributing to the occurrence of abnormal carbohydrate balance in patients with type 1 diabetes may be EBV infection. Based on the obtained results, we also found an increase in the percentage of T cells with the CD4+CD3+IL-17+ phenotype in patients with anti-VCA antibodies in the IgG class. Furthermore, the accompanying significantly higher concentrations of fructosamine and higher levels of HbA1c may confirm the involvement of IL-17 in the weakening of metabolism glucose and suggest that EBV infection is accompanied by an increase in IL-17 secretion.

## Figures and Tables

**Table 1:** Percentage and absolute values of selected subpopulations of lymphocytes in the study group and in the control group.

Parameter	Group	Mean	Median	Minimum	Maximum	SD	*p*-Value
CD3+ T lymphocytes (%)	Study group	71.93	71.18	60.69	83.83	6.24	0.74
	Control group	70.22	68.43	58.64	85.21	7.72	
CD3+ T lymphocytes (10^3^/mm^3^)	Study group	1.53	1.44	0.38	2.71	0.45	0.52
	Control group	1.79	1.63	1.07	2.73	0.50	
CD19+ B lymphocytes (%)	Study group	13.39	13.38	7.12	20.35	3.47	0.31
	Control group	11.92	12.38	5.82	15.73	2.80	
CD19+ B lymphocytes (10^3^/mm^3^)	Study group	0.28	0.30	0.06	0.47	0.09	0.43
	Control group	0.32	0.33	0.16	0.53	0.11	
CD3+CD4+ T lymphocytes (%)	Study group	39.30	39.79	20.90	50.36	6.31	0.29
	Control group	36.91	36.78	26.88	44.85	5.28	
CD3+CD4+ T lymphocytes (10^3^/mm^3^)	Study group	0.85	0.81	0.21	1.58	0.28	0.38
	Control group	0.95	0.82	0.53	1.52	0.34	
CD3+CD8+ T lymphocytes (%)	Study group	26.68	26.23	16.08	55.79	7.05	0.47
	Control group	26.17	25.13	17.40	36.55	5.22	
CD3+CD8+ T lymphocytes (10^3^/mm^3^)	Study group	0.58	0.54	0.17	1.13	0.23	0.79
	Control group	0.67	0.68	0.41	0.99	0.18	
CD3+CD4+/ CD3+CD8+ ratio	Study group	1.61	1.52	0.37	3.04	0.52	0.40
	Control group	1.47	1.39	0.83	2.40	0.39	
NK CD3-CD16+CD56+ (%)	Study group	12.21	10.42	2.10	25.19	5.57	0.71
	Control group	14.18	13.08	6.43	25.15	6.65	
NK CD3-CD16+CD56+ (10^3^/mm^3^)	Study group	0.27	0.26	0.04	0.60	0.14	0.63
	Control group	0.36	0.30	0.14	0.97	0.21	

**Table 2:** Percentage of T and B lymphocytes expressing the CD69 antigen in the study group and the control group.

Parameter	Group	Mean	Median	Minimum	Maximum	SD	*p*-Value
CD3+ CD69+ T lymphocytes (%)	Study group	3.72	3.15	1.25	9.23	1.86	0.123
	Control group	3.01	2.72	1.12	5.58	1.65	
CD19+ CD69+ B lymphocytes (%)	Study group	12.43	9.87	1.52	48.77	10.47	0.245
	Control group	9.79	7.53	2.49	20.96	6.28	
CD4+ CD69+ T lymphocytes (%)	Study group	4.53	3.72	0.95	10.20	2.55	0.029 *
	Control group	3.09	2.60	0.56	9.30	2.31	
CD8+ CD69+ T lymphocytes (%)	Study group	1.58	1.30	0.17	3.70	0.86	0.003 *
	Control group	0.96	0.77	0.15	2.87	0.73	
CD3+ CD25+ T lymphocytes (%)	Study group	17.60	16.22	7.93	28.60	4.91	0.004 *
	Control group	14.12	14.63	8.60	18.31	2.65	
CD19+ CD25+ B lymphocytes (%)	Study group	20.38	19.18	7.85	38.16	6.88	0.219
	Control group	19.87	19.77	11.24	31.29	5.22	
CD4+ CD25+ T lymphocytes (%)	Study group	29.26	28.73	15.29	47.83	7.40	0.044 *
	Control group	24.80	26.33	13.45	32.30	5.79	
CD8+ CD25+ T lymphocytes (%)	Study group	1.40	1.03	0.46	6.49	1.15	0.139
	Control group	1.05	0.92	0.40	2.19	0.55	

* Statistically significant *p* < 0.05.

**Table 3:** Evaluation of the percentage of CD3+CD4+ lymphocytes with intracellular expression of selected cytokines in the study group and the control group.

Parameter	Group	Mean	Median	Minimum	Maximum	SD	*p*-Value
CD3+ CD4+IFN-γ+ T lymphocytes (%)	Study group	1.38	0.94	0.12	7.26	1.54	0.236
	Control group	1.76	1.30	0.12	4.19	1.29	
CD3+ CD4+IL-2+ T lymphocytes (%)	Study group	2.99	2.32	0.11	11.83	2.80	0.187
	Control group	2.93	2.71	0.29	5.38	1.50	
CD3+ CD4+IL-4+ T lymphocytes (%)	Study group	0.40	0.36	0.11	1.19	0.24	0.004 *
	Control group	0.84	0.72	0.12	2.40	0.64	
CD3+ CD4+IL-10+ T lymphocytes (%)	Study group	0.17	0.13	0.10	0.80	0.12	0.312
	Control group	0.19	0.15	0.10	0.51	0.12	
CD3+ CD4+IL-17+ T lymphocytes (%)	Study group	0.37	0.33	0.11	1.33	0.28	0.411
	Control group	0.46	0.29	0.14	2.20	0.54	

* Statistically significant *p* < 0.05.

**Table 4:** Evaluation of anti-VCA antibodies in the IgM and IgG class and anti-EBNA-1 in the IgG class in the study group and the control group.

Parameter	Group	Mean	Median	Minimum	Maximum	SD	*p*-Value
VCA IgM (U/mL)	Study group	20.63	18.51	13.21	37.18	6.68	-
	Control group	–	–	–	–	–	
VCA IgG (U/mL)	Study group	65.97	51.49	16.88	252.76	53.29	0.364
	Control group	38.51	32.70	14.82	84.97	19.90	
EBNA-1 IgG (U/mL)	Study group	23.94	21.94	13.60	39.68	7.69	0.019 *
	Control group	34.16	37.58	15.49	43.20	9.72	

* Statistically significant *p* < 0.05.

**Table 5:** Percentages and absolute values of selected subpopulations of lymphocytes in patients with type 1 diabetes depending on the presence of anti-VCA antibodies in the IgM and IgG class and anti-EBNA-1 in the IgG class.

Parameter	Group	Mean	Median	Minimum	Maximum	SD	*p*-Value
CD3+ T lymphocytes (%)	VCA IgM (+)	71.79	71.17	60.69	83.83	7.23	0.109
	VCA IgM (−)	72.40	71.38	64.02	81.91	4.43	
	VCA IgG (+)	72.45	72.96	60.69	83.83	6.43	0.297
	VCA IgG (−)	71.13	70.41	62.31	82.84	5.83	
	EBNA-1 IgG (+)	71.03	70.78	60.69	83.83	7.01	0.147
	EBNA-1 IgG (−)	72.99	73.34	62.31	82.84	5.31	
CD3+ T lymphocytes (10^3^/mm^3^)	VCA IgM (+)	1.60	1.47	0.96	2.71	0.47	0.264
	VCA IgM (−)	1.41	1.48	0.38	2.02	0.39	
	VCA IgG (+)	1.58	1.52	0.38	2.71	0.47	0.144
	VCA IgG (−)	1.38	1.26	1.07	2.02	0.33	
	EBNA-1 IgG (+)	1.54	1.51	0.38	2.71	0.48	0.321
	EBNA-1 IgG (−)	1.56	1.38	1.07	2.58	0.41	
CD19+ B lymphocytes (%)	VCA IgM (+)	13.47	13.32	7.26	20.35	3.64	0.122
	VCA IgM (−)	13.18	13.38	7.12	18.66	3.20	
	VCA IgG (+)	13.01	13.10	7.12	20.35	3.41	0.302
	VCA IgG (−)	14.11	14.07	8.34	19.72	3.49	
	EBNA-1 IgG (+)	13.70	14.01	7.78	20.35	3.19	0.104
	EBNA-1 IgG (−)	13.02	13.32	7.12	19.72	3.70	
CD19+ B lymphocytes (10^3^/mm^3^)	VCA IgM (+)	0.29	0.30	0.13	0.47	0.08	0.210
	VCA IgM (−)	0.26	0.27	0.06	0.40	0.10	
	VCA IgG (+)	0.28	0.29	0.06	0.47	0.09	0.410
	VCA IgG (−)	0.28	0.29	0.13	0.40	0.09	
	EBNA-1 IgG (+)	0.30	0.30	0.06	0.47	0.09	0.108
	EBNA-1 IgG (−)	0.25	0.24	0.13	0.04	0.09	
CD3+CD4+ T lymphocytes (%)	VCA IgM (+)	39.18	39.92	20.90	50.36	7.44	0.212
	VCA IgM (−)	39.52	39.79	32.89	47.73	3.92	
	VCA IgG (+)	39.05	39.32	20.90	48.64	5.92	0.134
	VCA IgG (−)	40.05	39.92	30.78	50.36	7.30	
	EBNA-1 IgG (+)	39.84	39.92	30.25	48.64	4.97	0.120
	EBNA-1 IgG (−)	38.67	38.25	20.90	50.36	7.62	
CD3+CD4+ T lymphocytes (10^3^/mm^3^)	VCA IgM (+)	0.89	0.79	0.40	1.58	0.30	0.142
	VCA IgM (−)	0.78	0.81	0.21	1.07	0.22	
	VCA IgG (+)	0.87	0.81	0.21	1.58	0.28	0.219
	VCA IgG (−)	0.79	0.75	0.49	1.37	0.26	
	EBNA-1 IgG (+)	0.87	0.83	0.21	1.58	0.28	0.106
	EBNA-1 IgG (−)	0.82	0.76	0.40	1.37	0.26	
CD3+CD8+ T lymphocytes (%)	VCA IgM (+)	26.36	26.44	16.08	55.79	8.06	0.117
	VCA IgM (−)	26.57	25.84	16.34	38.32	5.68	
	VCA IgG (+)	27.90	28.21	16.34	55.79	7.63	0.033 *
	VCA IgG (−)	23.06	24.44	16.08	29.42	4.47	
	EBNA-1 IgG (+)	25.82	27.09	16.34	37.54	5.47	0.202
	EBNA-1 IgG (−)	27.04	26.05	16.08	55.79	8.53	
CD3+CD8+ T lymphocytes (10^3^/mm^3^)	VCA IgM (+)	0.61	0.55	0.31	1.13	0.24	0.114
	VCA IgM (−)	0.52	0.54	0.17	0.85	0.20	
	VCA IgG (+)	0.62	0.59	0.17	1.13	0.24	0.041 *
	VCA IgG (−)	0.45	0.45	0.31	0.78	0.14	
	EBNA-1 IgG (+)	0.57	0.55	0.17	0.94	0.21	0.147
	EBNA-1 IgG (−)	0.58	0.49	0.31	1.13	0.25	
CD3+CD4+/CD3+CD8+ ratio	VCA IgM (+)	1.65	1.54	0.37	3.04	0.59	0.216
	VCA IgM (−)	1.58	1.50	0.92	2.44	0.42	
	VCA IgG (+)	1.52	1.48	0.37	2.52	0.47	0.302
	VCA IgG (−)	1.87	1.78	1.05	3.04	0.59	
	EBNA-1 IgG (+)	1.62	1.53	1.06	2.52	0.43	0.155
	EBNA-1 IgG (−)	1.63	1.52	0.37	3.04	0.62	
NK CD3-CD16+CD56+ (%)	VCA IgM (+)	12.56	10.63	3.39	25.19	6.00	0.163
	VCA IgM (−)	11.58	10.15	2.10	18.26	4.88	
	VCA IgG (+)	12.12	10.21	2.10	25.19	5.89	0.143
	VCA IgG (−)	12.47	10.63	6.58	18.20	4.81	
	EBNA-1 IgG (+)	12.90	14.61	2.10	25.19	6.63	0.125
	EBNA-1 IgG (−)	11.42	9.89	5.93	18.26	4.16	
NK CD3-CD16+CD56+ (10^3^/mm^3^)	VCA IgM (+)	0.29	0.27	0.08	0.60	0.14	0.120
	VCA IgM (−)	0.23	0.19	0.04	0.48	0.14	
	VCA IgG (+)	0.26	0.25	0.04	0.60	0.15	0.169
	VCA IgG (−)	0.29	0.28	0.17	0.48	0.12	
	EBNA-1 IgG (+)	0.28	0.26	0.04	0.60	0.16	0.208
	EBNA-1 IgG (−)	0.25	0.26	0.11	0.48	0.11	

(+)— positive; (−)–negative; * statistically significant *p* < 0.05.

**Table 6:** Comparison of the percentage of lymphocytes with the expression of the CD69+ antigen in patients with type 1 diabetes in relation to the presence of anti-VCA antibodies in the IgM and IgG class and anti-EBNA-1 in the IgG class.

Parameter	Group	Mean	Median	Minimum	Maximum	SD	*p*-Value
CD3+ CD69+ T lymphocytes (%)	VCA IgM (+)	3.59	3.09	1.65	9.23	1.80	0.078
	VCA IgM (−)	3.86	3.28	1.25	8.14	1.95	
	VCA IgG (+)	4.03	3.68	1.65	9.23	1.95	0.093
	VCA IgG (−)	2.81	2.69	1.25	5.74	1.17	
	EBNA-1 IgG (+)	4.22	3.72	1.65	9.23	2.11	0.141
	EBNA-1 IgG (−)	3.06	2.82	1.25	5.74	1.22	
CD19+ CD69+ B lymphocytes (%)	VCA IgM (+)	12.56	8.91	1.52	48.77	10.45	0.079
	VCA IgM (−)	11.82	9.66	1.62	42.05	10.63	
	VCA IgG (+)	12.18	10.07	1.62	42.05	9.27	0.103
	VCA IgG (−)	12.43	6.17	1.52	48.77	13.22	
	EBNA-1 IgG (+)	13.14	11.05	2.10	42.05	9.45	0.089
	EBNA-1 IgG (−)	11.22	6.11	1.52	48.77	11.58	
CD4+ CD69+ T lymphocytes (%)	VCA IgM (+)	4.72	3.72	1.41	10.20	2.82	0.116
	VCA IgM (−)	4.13	3.50	0.95	7.96	2.14	
	VCA IgG (+)	4.31	3.59	0.95	10.20	2.19	0.217
	VCA IgG (−)	4.85	3.64	1.41	10.13	3.36	
	EBNA-1 IgG (+)	4.65	4.51	0.95	10.20	2.25	0.269
	EBNA-1 IgG (−)	4.29	3.37	1.35	10.13	2.88	
CD8+ CD69+ T lymphocytes (%)	VCA IgM (+)	1.72	1.28	0.43	3.70	0.98	0.077
	VCA IgM (−)	1.39	1.31	0.17	2.87	0.62	
	VCA IgG (+)	1.42	1.23	0.17	3.25	0.78	0.029 *
	VCA IgG (−)	1.97	1.68	1.15	3.70	0.91	
	EBNA-1 IgG (+)	1.58	1.24	0.43	3.25	0.81	0.069
	EBNA-1 IgG (−)	1.57	1.31	0.17	3.70	0.91	
CD3+ CD25+ T lymphocytes (%)	VCA IgM (+)	17.09	15.47	7.93	27.02	4.57	0.122
	VCA IgM (−)	18.19	17.40	9.39	28.60	5.32	
	VCA IgG (+)	17.64	15.89	9.39	28.60	4.42	0.099
	VCA IgG (−)	17.35	18.70	7.93	27.57	5.97	
	EBNA-1 IgG (+)	18.39	16.77	13.29	28.60	4.45	0.178
	EBNA-1 IgG (−)	16.67	15.47	7.93	27.57	5.23	
CD19+ CD25+ B lymphocytes (%)	VCA IgM (+)	20.59	19.96	11.91	30.00	5.47	0.129
	VCA IgM (−)	20.08	17.75	7.85	38.16	8.42	
	VCA IgG (+)	20.70	18.64	11.35	38.16	7.05	0.143
	VCA IgG (−)	19.64	19.96	7.85	28.93	6.42	
	EBNA-1 IgG (+)	20.45	18.91	11.92	31.62	5.74	0.147
	EBNA-1 IgG (−)	20.29	19.96	7.85	38.16	7.96	
CD4+ CD25+ T lymphocytes (%)	VCA IgM (+)	28.26	28.26	15.29	44.60	6.66	0.096
	VCA IgM (−)	30.38	29.85	18.25	47.83	8.20	
	VCA IgG (+)	29.79	29.24	18.80	44.60	6.25	0.154
	VCA IgG (−)	27.69	26.76	15.29	47.83	9.55	
	EBNA-1 IgG (+)	30.56	29.73	21.67	44.6	6.42	0.312
	EBNA-1 IgG (−)	27.61	27.92	15.29	47.83	8.11	
CD8+ CD25+ T lymphocytes (%)	VCA IgM (+)	1.42	1.11	0.46	4.09	0.91	0.088
	VCA IgM (−)	1.39	0.96	0.56	6.49	1.43	
	VCA IgG (+)	1.40	1.02	0.4	6.49	1.26	0.073
	VCA IgG (−)	1.42	1.21	0.65	2.85	0.76	
	EBNA-1 IgG (+)	1.07	0.91	0.46	2.60	0.56	0.042 *
	EBNA-1 IgG (−)	1.82	1.33	0.65	6.49	1.51	

(+)— positive; (−)—negative; * statistically significant *p* < 0.05.

**Table 7:** Evaluation of the percentage of CD3+CD4+ T lymphocytes with intracellular expression of selected cytokines in patients with type 1 diabetes in relation to the presence of anti-VCA antibodies in the IgM, IgG and anti-EBNA-1 in the IgG class.

Parameter	Group	Mean	Median	Minimum	Maximum	SD	*p*-Value
CD3+ CD4+IFN-γ+ T lymphocytes (%)	VCA IgM (+)	1.36	1.02	0.12	6.47	1.46	0.087
	VCA IgM (−)	1.36	0.76	0.31	7.26	1.71	
	VCA IgG (+)	1.34	1.08	0.12	7.26	1.39	0.302
	VCA IgG (−)	1.39	0.65	0.19	6.47	1.87	
	EBNA-1 IgG (+)	1.423	1.10	0.14	7.26	1.51	0.078
	EBNA-1 IgG (−)	1.29	0.75	0.12	6.47	1.59	
CD3+ CD4+IL-2+ T lymphocytes (%)	VCA IgM (+)	3.16	2.89	0.12	11.83	2.93	0.069
	VCA IgM (−)	2.55	1.10	0.11	6.70	2.59	
	VCA IgG (+)	3.19	3.11	0.11	6.70	2.54	0.110
	VCA IgG (−)	2.44	1.05	0.12	11.83	3.29	
	EBNA-1 IgG (+)	3.51	3.20	0.12	6.45	2.32	0.118
	EBNA-1 IgG (−)	2.43	0.96	0.11	11.83	3.12	
CD3+ CD4+IL-4+ T lymphocytes (%)	VCA IgM (+)	0.39	0.37	0.11	0.81	0.18	0.094
	VCA IgM (−)	0.43	0.30	0.11	1.19	0.35	
	VCA IgG (+)	0.42	0.39	0.11	1.19	0.26	0.103
	VCA IgG (−)	0.36	0.32	0.11	0.88	0.20	
	EBNA-1 IgG (+)	0.41	0.38	0.11	0.86	0.22	0.159
	EBNA-1 IgG (−)	0.39	0.33	0.11	1.19	0.27	
CD3+ CD4+IL-10+ T lymphocytes (%)	VCA IgM (+)	0.18	0.12	0.10	0.80	0.14	0.210
	VCA IgM (−)	0.15	0.13	0.10	0.34	0.07	
	VCA IgG (+)	0.19	0.18	0.10	0.80	0.13	0.011 *
	VCA IgG (−)	0.12	0.10	0.10	0.16	0.02	
	EBNA-1 IgG (+)	0.20	0.18	0.10	0.80	0.15	0.033 *
	EBNA-1 IgG (−)	0.13	0.10	0.10	0.23	0.04	
CD3+ CD4+IL-17+ T lymphocytes (%)	VCA IgM (+)	0.38	0.33	0.11	1.33	0.30	0.113
	VCA IgM (−)	0.36	0.32	0.12	1.02	0.24	
	VCA IgG (+)	0.39	0.35	0.11	1.02	0.20	0.030 *
	VCA IgG (−)	0.34	0.15	0.12	1.33	0.40	
	EBNA-1 IgG (+)	0.36	0.33	0.11	1.02	0.22	0.099
	EBNA-1 IgG (−)	0.38	0.32	0.12	1.33	0.33	

(+)— positive; (−)—negative; * statistically significant *p* < 0.05.

**Table 8:** Evaluation of selected parameters of carbohydrate balance in patients with type 1 diabetes in relation to the presence of anti-VCA antibodies in the IgM, IgG and anti-EBNA-1 in the IgG class.

Parameter	Group	Mean	Median	Minimum	Maximum	SD	*p*-Value
Fructosamine (µmol/L)	VCA IgM (+)	501.74	458.00	257.00	962.00	179.38	0.089
	VCA IgM (−)	541.94	576.50	220.00	921.00	181.97	
	VCA IgG (+)	596.37	600.00	340.00	962.00	165.98	<0.001 *
	VCA IgG (−)	370.93	359.50	220.00	520.00	88.68	
	EBNA-1 IgG (+)	588.79	580.00	340.00	962.00	173.95	0.015 *
	EBNA-1 IgG (−)	459.45	415.00	220.00	921.00	165.03	
HbA1c level (%)	VCA IgM (+)	8.69	8.70	5.00	13.80	2.11	0.073
	VCA IgM (−)	9.71	9.70	6.20	15.00	2.24	
	VCA IgG (+)	9.62	9.50	5.80	15.00	2.20	0.017 *
	VCA IgG (−)	7.80	8.10	5.00	10.00	1.60	
	EBNA-1 IgG (+)	9.42	8.90	5.80	15.00	2.42	0.066
	EBNA-1 IgG (−)	8.75	8.85	5.00	13.80	1.95	

(+)— positive; (−)—negative; * statistically significant *p* < 0.05.

**Table 9:** Characteristics of selected parameters and blood-morphology parameters of patients from the study and control groups.

Parameter		Study Group (*n* = 43)	Control Group (*n* = 20)
Sex	Girls	21 (48.8%)	8 (40%)
	Boys	22 (51.2%)	12 (60%)
Age	Mean ± SD	13.59 ± 3.55	11.02 ± 2.49
	Median (minimum–maximum)	14 (3.9–18.0)	10.85 (7.75–14.4)
BMI	Mean ± SD	20.13 ± 3.63	15.83 ± 1.67
	Median (minimum–maximum)	19.94 (11.19–31.31)	15.9 (12.72–18.5)
Leukocytosis (10^3^/µL)	Mean ± SD	6.59 ± 2.05	6.59 ± 0.99
	Median (minimum–maximum)	5.85 (3.63–12.02)	6.22 (5.15–8.64)
Lymphocytosis (10^3^/µL)	Mean ± SD	2.14 ± 0.56	2.64 ± 0.74
	Median (minimum–maximum)	2.03 (0.54–3.62)	2.39 (1.68–4.34)
Monocytosis (10^3^/µL)	Mean ± SD	0.58 ± 0.38	0.57 ± 0.12
	Median (minimum–maximum)	0.52 (0.20–2.63)	0.55 (0.39–0.80)
Neutrocytosis (10^3^/µL)	Mean ± SD	3.60 ± 2.01	3.16 ± 0.89
	Median (minimum–maximum)	2.93 (0.35–9.62)	3.16 (1.27–4.60)
Erythrocytes (10^6^/µL)	Mean ± SD	4.95 ± 0.42	4.71 ± 0.36
	Median (minimum-maximum)	4.89 (4.26–6.02)	6.64 (4.23–5.61)
Hemoglobin (g/dL)	Mean ± SD	14.26 ± 1.25	13.29 ± 0.93
	Median (minimum–maximum)	14.00 (11.90–17.10)	13.20 (11.90–16.00)
Hematocrit (%)	Mean ± SD	40.37 ± 3.33	37.99 ± 2.73
	Median (minimum–maximum)	40.70 (32.90–48.50)	38.00 (33.70–46.00)
Platelets (10^3^/µL)	Mean ± SD	251.13 ± 46.36	326.65 ± 58.43
	Median (minimum–maximum)	255.00 (154.00–361.00)	321.00 (241.00–443.00)

## Data Availability

Due to privacy and ethical concerns, the data that support the findings of this study are available on request from the first author (M.K.).

## Abbreviations

APC	antigen-resenting cells
CD	cluster of differentiation
CMV	cytomegalovirus
DM1	diabetes mellitus type 1
DM1A	diabetes mellitus type 1A
DM1B	diabetes mellitus type 1B
EBNA	Epstein–Barr nuclear antigen
EBV	Epstein–Barr virus
EDTA	ethylenediaminetetraacetate
FITC	fluorescein isothiocyanate
FSC	forward-scatter channel
HbA1c	glycated hemoglobin; hemoglobin A1c
IFN-γ	interferon-γ
IL	interleukin
NK	natural killer cells
PBMC	peripheral mononuclear blood cells
PBS	phosphate-buffered saline
SSC	side-scatter channel
TNF	tumor-necrosis factor
Treg	regulatory T cells
VCA	viral-capsid antigen complex
